# Supplementary material for: The PSI Domain of the MET Oncogene Encodes a Functional Disulfide Isomerase Essential for the Maturation of the Receptor Precursor
Source: Int J Mol Sci. 2022 Oct 17;23(20):12427. doi: 10.3390/ijms232012427 (PMC9604360; doi:10.3390/ijms232012427)
Supplement: Supplementary file 1 [file ijms-23-12427-s001.zip › ijms-1928087-supplementary.pdf]

Supplementary Table S1: List of antibodies used in the study. WB: Western Blot; IF: Immunofluorescence; FACS: flow cytometry.

Name	Reference	Company	Dilution	Species
Phospho-MET-Y1234/1235	3077	Cell Signalling	1/500 WB, 1/500 IF, 1/200 FACS	Rabbit
Phospho-AKT (Ser473)	9271	Cell Signalling	1/1000 WB 1/200 IF	Rabbit
MET	3148	Cell Signalling	1/1000 WB	Mouse
MET-PE conjugated	95106	R&D	1/20 FACS	Mouse
p44/42 MAPK (Erk1/2)	9102	Cell Signalling	1/1000 WB	Rabbit
Phospho-p44/42 MAPK (Erk1/2) (Thr202/Tyr204)	9101	Cell Signalling	1/1000 WB	Rabbit
AKT	9272	Cell Signalling	1/1000 WB	Rabbit
GM130	ab52649	Abcam	1/250 IF	Rabbit
VIMENTIN	ab20346	ab20346	1μg/million of cells FACS	Mouse
GAPDH	2118	Cell Signalling	1/1000 WB	Rabbit
58K-Golgi	ab27043	Abcam	1/250 IF	Mouse
Beta-ACTIN-RHP	ab49900	Abcam	1/10000 WB	Mouse
H3-HRP	ab21054	Abcam	1/5000 WB	Rabbit

Supplementary Table S2: Primers used for site-directed mutagenesis. The DNA template is the Addgene 37560 plasmid containing the human MET_wt cDNA.

Name	Sequence	Tm	Purpose
CXXC_1_F	caagccctcTCTGCCCCACCCTTTGTT	64	First CXXC substitution to AXXA
CXXC_1_R	actggcggaCTGGAAATGTCTGCAGCC	64	First CXXC substitution to AXXA
CXXC_2_F	tgggcccacGACAAATGTGTGCGATCGG	65	Second CXXC substitution to AXXA
CXXC_2_F	gccagcctgAACAAAGGGTGGGGCAGA	65	Second CXXC substitution to AXXA
CXXC_1_2_F	ccctttgttcaggctggctgggcccacGACAAATGTGTGCGATCG	62	CXXC1 and CXXC2 to AXXA1 and AXXA2
CXXC_1_2_R	tggggcagagagggcttgactggcggaCTGGAAATGTCTGCAGCC	62	CXXC1 and CXXC2 to AXXA1 and AXXA2
PSI_F	TGTCTGCCTGCAATCTAC	61	Complete deletion of the PSI domain, from pLenti-MET_GFP
PSI_R	CAAGCCATTCAATGGGATC	61	Complete deletion of the PSI domain, from pLenti-MET_GFP
